# The relationship between social media fatigue and online trolling behavior among college students: the mediating roles of relative deprivation and hostile attribution bias

**DOI:** 10.3389/fpsyg.2024.1495235

**Published:** 2025-01-06

**Authors:** Lexin Huang, Liangkun Chen, Suwei Ma

**Affiliations:** ^1^School of Philosophy and Social Development, South China Normal University, Guangzhou, China; ^2^School of Marxism, Jiangnan University, Wuxi, China

**Keywords:** social media fatigue, online trolling behavior, relative deprivation, hostile attribution bias, mediating effects

## Abstract

Based on the General Aggression Model (GAM), this study explores the relationship between social media fatigue and online trolling behavior among Chinese college students, focusing on the mediating roles of relative deprivation and hostile attribution bias as key affective and cognitive mechanisms proposed by GAM. Using a cross-sectional survey design, data were collected from 349 college students from Guangdong via an online questionnaire. Key variables, including social media fatigue, relative deprivation, and hostile attribution bias, were measured using validated scales: the SNS Fatigue Questionnaire, the Personal Relative Deprivation Scale, the Word Sentence Association Paradigm for Hostility, and the revised Global Assessment of Internet Trolling. Structural equation modeling (SEM) was employed to test the hypothesized relationships and mediating effects. The results indicate that social media fatigue is positively associated with online trolling behavior. Furthermore, relative deprivation and hostile attribution bias serve as significant mediating mechanisms in this relationship, forming a chain mediation model. These findings suggest that when users experience social media fatigue, they may feel deprived relative to others, which can lead to a hostile interpretation of others’ behaviors, thereby increasing the likelihood of engaging in trolling. The study highlights the importance of understanding psychological factors that contribute to negative online behaviors and offers insights into potential intervention strategies to mitigate such behaviors by addressing the underlying psychological mechanisms.

## 1 Introduction

With the rapid development of the Internet and social media, social networks have become an integral part of people’s daily lives. According to the 54th “Statistical Report on China’s Internet Development” released by the China Internet Network Information Center (CNNIC), as of June 2024, the number of Internet users in China reached 1.09967 billion, with an Internet penetration rate of 78.0% (Times, n.d.). Surveys indicate that 65% of Chinese students spend over 3 h online daily (Xinhua, n.d.). However, alongside the widespread use of social media, some negative phenomena have gradually emerged, among which online trolling behavior and social media fatigue are particularly noteworthy.

Online trolling behavior is essentially a form of aggressive behavior. Buckels et al. define online trolling as the deliberate posting of offensive, provocative, or hateful comments on the Internet to provoke disputes, harm others, or gain amusement (Buckels et al., 2014). This definition clearly identifies the aggressive nature of online trolling behavior. From the perspective of the General Aggression Model (GAM), online trolling behavior aligns with the characteristics of aggressive behavior: it involves intentional harm to others and typically stems from negative emotions and cognitions (Allen et al., 2018). Furthermore, online trolling behavior also fits the definition of cyber-aggressive behavior, which refers to intentional and targeted aggressive acts against individuals or groups through electronic or digital media (Mehari et al., 2014).

In the context of cyber-aggressive behavior, it is important to understand the relationship between online trolling and cyberbullying, as the latter has been extensively studied in the literature. While both represent forms of aggressive online behavior, they emphasize different characteristics. Cyberbullying refers to intentional and repeated harmful behavior targeting specific internet users through electronic devices (Hinduja and Patchin, 2010), often involving clear social identities and explicit aggressive intentions. Although some researchers suggest that trolling can be considered a form of cyberbullying (Morrissey, 2010; Griffiths, 2014), the key distinction lies in their behavioral patterns and motivations: trolling primarily focuses on deception and meaningless disruption, with perpetrators often concealing their true motivations, while cyberbullying typically involves more direct and targeted aggression with clearer intentions (Aydın et al., 2021). Despite these differences, both behaviors may share common psychological mechanisms, such as moral disengagement and hostile attribution bias (Kowalski et al., 2014). Understanding this relationship helps position our investigation of trolling behavior in the broader context of online aggression while maintaining focus on its unique characteristics, particularly how its deceptive and disruptive nature may relate to social media fatigue and psychological states.

Research indicates that online trolling is prevalent among college students (Case and King, 2017; Hong and Cheng, 2018). The 2017 Ofcom survey examining adult media behavior revealed that while 1% of British internet users encountered trolling in the preceding 12 months, the figure was significantly higher (5%) among young adults between 16 and 24 years old (Ofcom, 2017). In another study, researchers examined cyberbullying and victimization among Turkish university students. The results showed that experiencing bullying as a victim in cyberspace predicted a 23% probability of becoming an online troll (Akbulut and Eristi, 2011). Howard et al.’s study focused on college students and investigated the demographic and psychosocial predictors of malicious trolling on social media. It concluded that male gender, a higher need for social media participation, and a tendency to engage in downward social comparisons are significant predictors of trolling behavior (Howard et al., 2019). In the Chinese context, Studies have found that Chinese trolling often manifests as collective action rather than individual behavior, what particularly distinguishes Chinese online trolling is its fluid nature, where participants dynamically shift between roles—moving fluidly between being trolls, targets, and observers (Sun and Fichman, 2018).

Social media fatigue refers to the feeling of exhaustion, boredom, and weariness that users develop while using social media, influenced by personal, platform, and social factors (Bright et al., 2015). This feeling leads to reduced frequency and duration of social media use, and even withdrawal from social media platforms. Research shows that nearly 25% of social media users are beginning to experience fatigue, and 31% of enthusiasts report starting to feel bored with social networks (Insurance-Canada.ca, 2011).

There is a potential association between social media fatigue and online trolling behavior. First, from the perspective of stress-coping theory, social media fatigue may lead to negative emotions and stress in users, and online trolling behavior may become a maladaptive way of coping with this stress (Lazarus and Folkman, 1984). When users feel fatigued and bored, they may be more inclined to vent negative emotions to gain momentary psychological satisfaction, thereby increasing the likelihood of online trolling behavior. Second, according to the Limited Capacity Model (LCM), Social media fatigue may stem from information overload (Lang, 2000). When users face large amounts of information, their cognitive resources may be depleted, leading to decreased emotional regulation ability and increased likelihood of aggressive behavior, including online trolling. Moreover, Social media fatigue may reduce users’ self-control ability. According to Ego Depletion Theory, prolonged use of social media may deplete an individual’s self-control resources (Baumeister et al., 2007). When these resources are exhausted, users may find it more difficult to suppress their impulses and are more likely to post aggressive comments.

However, current academic research on the relationship between social media fatigue and online trolling behavior is relatively scarce. Most studies focus on exploring the causes and influencing factors of social media fatigue (Zhang et al., 2016), or analyzing the motivations and characteristics of online trolling behavior (Cheng et al., 2017), but few studies have systematically examined the combination of the two. Therefore, this study aims to explore the mechanism by which social media fatigue influences online trolling behavior. By reviewing existing literature, constructing theoretical models, and conducting empirical research, we hope to reveal the intrinsic connection between social media fatigue and online trolling behavior, providing theoretical basis and practical insights for understanding and mitigating these two negative phenomena.

## 2 Literature reviews and hypotheses development

### 2.1 Social media fatigue and online trolling behavior

The widespread use of social media has led to user fatigue and aggressive behavior, a phenomenon that has garnered significant attention. Studies have shown that social media-related stressors, such as information overload and social comparison, directly contribute to users’ emotional exhaustion, subsequently leading to social media fatigue (Lim and Choi, 2017). Prolonged use of social networks significantly increases users’ psychological stress, not only resulting in emotional fatigue but also potentially prompting aggressive behavior when faced with an unfair online environment (Maier et al., 2012a). When users are unable to effectively cope with the negative emotions brought about by social media, they often resort to aggressive behavior as a means of releasing this stress (Zhang et al., 2012). Research has also indicated that technological dependence significantly impacts social behavior, with extended social media use leading not only to deterioration in interpersonal relationships but also to an increase in aggressive behavior (Rosen, 2013). The sense of exclusion on social media is considered a crucial factor in triggering aggressive behavior among users, particularly when individuals feel excluded or ignored by others (Tufekci, 2008). Furthermore, social media fatigue is viewed as one of the primary reasons users either cease using platforms or exhibit aggressive responses (Maier et al., 2012b).

Privacy concerns have also been identified as a potential factor in precipitating aggressive behavior. Users who have been using social media for extended periods may experience heightened aggressive reactions due to dissatisfaction with privacy issues (Kim and Kankanhalli, 2009). Studies have found that the discrepancy between users’ self-presentation on social media and their real-life personas often leads to frustration, which further exacerbates aggressive behavior (Gibbs et al., 2006). In the face of persistent stress from social media, users lacking effective coping strategies are more prone to displaying aggressive behavior (Compas et al., 2001). Ravindran et al. posit that social media fatigue is a multidimensional phenomenon, encompassing boredom, disappointment, and loss of interest in social media use (Ravindran et al., 2014). Wu et al. further categorize social media fatigue into two dimensions: loss of interest and exhaustion (Wu et al., 2022). The factors contributing to social media fatigue are diverse. Lee et al.’s research identified system feature overload, information overload, and social overload as primary causes of social media fatigue (Lee et al., 2016). Hsu et al. point out that the abundance of advertisements and irrelevant information on social media platforms also intensifies users’ sense of fatigue (Hsu et al., 2023). Additionally, Qiu et al.’s study demonstrates that frequent exposure to antisocial media content not only increases users’ social media fatigue but also exacerbates their hostile attribution bias (Qiu et al., 2024). Given these findings, it is evident that social media fatigue may be significantly associated with online trolling behavior. Therefore, this study proposes *Hypothesis 1*: Social media fatigue is positively associated with online trolling behavior.

### 2.2 The mediating role of relative deprivation

Relative deprivation is a psychological state that arises from social comparison, potentially triggering a range of negative emotions and behaviors when individuals perceive themselves to be unfairly disadvantaged. Research indicates that relative deprivation can significantly increase an individual’s propensity for online aggressive behavior by eliciting negative emotions such as anger and resentment (Smith et al., 2012; Greitemeyer and Sagioglou, 2017). Further studies suggest that relative deprivation is not only associated with aggressive behavior in real life but can also propagate through social networks, exacerbating interpersonal hostility (Greitemeyer and Sagioglou, 2018). In environments characterized by social structural inequality, individuals’ sense of relative deprivation often leads to higher levels of hostility and violent tendencies, particularly in situations of severe social stratification (Baron, 2003; DeCelles and Norton, 2016).

Studies show that social comparison is one of the core mechanisms in the formation of relative deprivation. When individuals compare themselves to those of higher social status, feelings of dissatisfaction often arise. If this dissatisfaction is not effectively mitigated, it may evolve into aggressive behavior (Wills, 1981). Moreover, the impact of relative deprivation on mental health is significant. Research indicates that relative deprivation is not only associated with depressive symptoms but may also lead individuals to choose riskier behaviors when faced with stress, further exacerbating the occurrence of online aggressive behavior (Beshai et al., 2017). Other studies have found that an individual’s sense of deprivation affects their risk assessment, making them more prone to engaging in aggressive and antisocial behaviors (Mishra and Novakowski, 2016). Research points out that a decrease in subjective social status is closely related to an increase in relative deprivation, which further prompts individuals to adopt hostile behaviors (Callan et al., 2015). Furthermore, relative deprivation can spread through social networks, leading to broader social unrest and collective aggressive behavior. Research indicates that physical and situational inequalities may lead to intergroup hostility and, in some cases, trigger collective aggressive behavior (DeCelles and Norton, 2016). Additionally, relative deprivation promotes individual participation in online aggressive behavior through psychological mechanisms such as rumination and moral disengagement, forming a vicious cycle (Crosby, 1976).

Concurrently, social media fatigue exhibits positive associations with relative deprivation. Research has shown that social media use can reduce relative deprivation by enhancing personal social capital and facilitating effective communication with influential others (Cho, 2014). Through structural equation modeling analysis, social capital was found to play a crucial mediating role in this process, meaning that social media users can, to some extent, alleviate negative feelings caused by social inequality by expanding and strengthening their social networks (Cho, 2014). However, another study points out that passive use of social media, such as merely browsing content posted by others without interaction, can actually exacerbate relative deprivation (Gkinopoulos et al., 2023). This passive usage often triggers negative social comparisons, leading users to experience more negative emotions and relative deprivation (Gkinopoulos et al., 2023). Further research suggests that the psychological stress brought about by impression management, privacy concerns, and maintaining interpersonal relationships may intensify relative deprivation among social media users during usage (Lee et al., 2019). Although social media aims to promote social interaction, these stressors may cause users to experience more psychological fatigue and negative emotions, thereby exacerbating relative deprivation (Lee et al., 2019). In summary, while social media has the potential to alleviate relative deprivation, its actual effect largely depends on usage patterns and the user’s psychological state.

Moreover, studies indicate that social media use may lead to feelings of envy among users, thereby increasing relative deprivation (Tandoc et al., 2015). Particularly when browsing others’ successes and happy lives, users are more likely to feel their own inadequacies, intensifying psychological imbalance (Tandoc et al., 2015). Frequent use of social media, especially frequent engagement in social comparison, may trigger a series of negative emotions, including depression and anxiety, which are closely related to relative deprivation (Verduyn et al., 2017). Negative social comparisons on social media are considered one of the important factors leading to depressive symptoms, which in turn further exacerbate relative deprivation (Feinstein et al., 2013). Research has also found that although social media provides opportunities to connect with others, frequent use can lead to a decline in personal well-being, partly due to the enhancement of relative deprivation (Kross et al., 2013). Therefore, this study proposes *Hypothesis 2*: Relative deprivation serves as a mediating mechanism in the association between social media fatigue and online trolling behavior.

### 2.3 The mediating role of hostile attribution bias

Hostile Attribution Bias (HAB) is closely associated with aggressive behavior. Research indicates that when individuals tend to interpret others’ ambiguous behaviors as hostile in social situations, this cognitive bias leads to higher aggressive responses. According to the Social Information Processing model, hostile attribution bias is one of the key factors in the development of aggressive behavior, particularly playing a crucial role in reactive aggression (Dodge, 2006). Studies have found that interventions targeting hostile attribution bias can, to some extent, reduce aggressive behavior, although the effects of such interventions are often limited (Hudley and Graham, 1993). Recent research has explored the use of more implicit training methods to alter hostile attribution bias and reduce aggressive behavior. For instance, training in facial expression recognition to enhance individuals’ ability to make positive judgments in ambiguous situations has shown promising results. This training not only reduced hostile attribution bias but also effectively lowered self-reported aggression and anger (Penton-Voak et al., 2013). Similar results were validated in high-risk adolescent groups, although these findings were not fully replicated in clinical samples (Hiemstra et al., 2019).

Further studies demonstrate that training adolescents to make more positive interpretations in ambiguous social situations can reduce their reactive aggressive behavior, with less impact on proactive aggression (Van Bockstaele et al., 2020). This finding aligns with earlier research conclusions that hostile attribution bias is primarily associated with reactive aggression (Orobio de Castro et al., 2002; de Castro et al., 2005). Additionally, online cognitive intervention measures have effectively reduced hostile attribution bias and decreased cyberbullying and aggressive driving behaviors (Osgood et al., 2021). Hostile attribution bias refers to the cognitive tendency of individuals to interpret ambiguous behaviors of others as hostile (Dodge, 2006). The General Aggression Model (GAM) proposed by Anderson and Bushman suggests that situational factors (such as exposure to antisocial content) may influence behavior by activating aggressive cognitions (like hostile attribution bias) (Anderson and Bushman, 2002). Crick and Dodge’s Social Information Processing model also supports this view, emphasizing the crucial role of cognitive processes in social behavior (Crick and Dodge, 1994). Research indicates a close association between social media fatigue and hostile attribution bias. Zhu et al.’s longitudinal study confirmed the relationship between negative online experiences, hostile thinking, and cyberbullying behavior (Zhu et al., 2023). Cover argues that large-scale online hostile behavior not only undermines users’ sense of self-worth but also positions victims as “ungrievable subjects” (Cover, 2022). This experience may further exacerbate users’ hostile attribution bias.

However, Mitchell et al.’s research suggests that empathy may play a buffering role in this process (Mitchell et al., 2021). Individuals with higher levels of empathy are less likely to engage in aggressive behavior after exposure to negative content. This provides a possible intervention direction for mitigating social media fatigue and hostile attribution bias. Faced with the negative impacts of social media fatigue and hostile attribution bias, users adopt different coping strategies. Teng et al. found that some users choose to reduce their frequency of social media use or temporarily exit certain platforms (Teng et al., 2022). Cover’s research indicates that many users tend to cope with these challenges through mutual support and self-care (Cover, 2022). This mutual support is manifested not only in personal protection but also in using alternative platforms to share strategies and support, as well as caring for the broader digital culture. Notably, Liu and Guo point out that social media fatigue may lead users to engage in surface acting, i.e., concealing their true emotions (Liu and Guo, 2017). This behavior may further exacerbate hostile attribution bias. Therefore, this study proposes *Hypothesis 3*: Hostile attribution bias mediates the relationship between social media fatigue and online trolling behavior.

### 2.4 The chain mediating effect of relative deprivation and hostile attribution bias

Previous research has demonstrated that relative deprivation significantly increases individuals’ tendency towards hostile attribution, subsequently leading to higher levels of aggressive behavior. Relative deprivation typically elicits negative emotions such as anger, which further provoke aggressive behavior directed at the perceived source of deprivation (Smith et al., 2012). Studies have found that individuals’ sense of relative deprivation can significantly enhance hostility and aggression towards others (Greitemeyer and Sagioglou, 2017). Relative deprivation not only affects individuals’ emotional responses but also exacerbates aggressive behavior through social cognitive processes such as hostile attribution. According to the Social Information Processing (SIP) model, hostile attribution bias is a key factor influencing reactive aggressive behavior (Crick and Dodge, 1994), hostile attribution bias has been found to significantly predict changes in individuals’ displaced aggressive behavior. Further research indicates a bidirectional relationship between relative deprivation and hostile attribution. Not only does relative deprivation enhance hostile attribution, but hostile attribution may also, in turn, reinforce relative deprivation, creating a vicious cycle (Crick and Dodge, 1994; Smith et al., 2012). Additionally, hostile attribution may further intensify aggressive behavior by influencing individuals’ moral disengagement. Studies show that relative deprivation can enhance moral disengagement by lowering individuals’ moral standards, making them more prone to engaging in aggressive behavior (Cen et al., 2022). Based on these findings, we propose *Hypothesis 4*: Relative deprivation and hostile attribution bias serially mediate the relationship between social media fatigue and online trolling behavior.

Based on the preceding literature review, this study adopts the General Aggression Model (GAM) as the overarching theoretical framework to integrate the complex relationships among social media fatigue, relative deprivation, hostile attribution bias, and online trolling behavior. GAM provides a comprehensive framework that can effectively explain how situational factors (such as social media fatigue) influence aggressive behavior through multiple pathways involving cognition, affect, and arousal. The selection of GAM as our theoretical cornerstone rests on several compelling considerations. Primarily, the model’s sophisticated treatment of personal-situational factor interactions mirrors the dynamic nature of social media engagement, where individual psychological conditions, such as fatigue, converge with platform-specific features to shape behavioral outcomes. Additionally, GAM’s dual recognition of cognitive mechanisms (manifested in hostile attribution bias) and affective pathways (expressed through relative deprivation) provides robust theoretical underpinning for our proposed chain mediation framework. The model’s demonstrated versatility in digital contexts further validates its selection, as evidenced by its successful application in recent investigations examining various manifestations of cyber aggression (Kowalski et al., 2014; Wright, 2020). Moreover, GAM’s process-centric orientation illuminates how initial states of social media fatigue can transform into aggressive behaviors through sequential cognitive and emotional mechanisms, particularly when considering the diminished self-regulatory resources characteristic of fatigue states. The model also accounts for both immediate situational factors and the development of aggressive knowledge structures over time, which is crucial for understanding how prolonged social media fatigue might contribute to the formation of hostile attribution patterns and ultimately lead to trolling behavior.

The hypothesized relationships align well with the theoretical framework of GAM. Specifically, H1 corresponds to GAM’s proposition about how situational factors relate to aggressive outcomes. H2 and H3 reflect the affective and cognitive routes proposed by GAM, respectively. H4 mirrors GAM’s integrated process model where multiple internal states work in concert to influence aggressive behavior. This theoretical alignment provides additional support for our hypothesized relationships and offers a unified framework for understanding the complex mechanisms underlying online trolling behavior.

In summary, guided by the General Aggression Model (GAM), we identify social media fatigue, relative deprivation, and hostile attribution bias as significant factors influencing online trolling behavior. According to GAM, these factors represent both situational inputs (social media fatigue) and internal states (relative deprivation and hostile attribution bias) that collectively predict aggressive behavior (online trolling). To empirically test our GAM-based hypotheses, this study constructs a structural equation model (SEM) with social media fatigue as the independent variable, online trolling behavior as the dependent variable, and relative deprivation and hostile attribution bias as sequential mediating variables that represent the affective and cognitive routes specified in GAM (Lei and Wu, 2007).

## 3 Method

### 3.1 Participants

This study recruited college students from higher education institutions in Guangdong Province using the sampling services provided by “Wenjuanxing,” an online survey platform similar to Amazon Mechanical Turk. A total of 465 questionnaires were distributed. To ensure data quality, several attention check questions were included (e.g., “Please select ‘Agree’ for this question”), and questionnaires were deemed invalid if the response time fell outside ±3 standard deviations, if the same option was excessively selected, or if responses showed a repetitive pattern. After excluding invalid questionnaires, 349 valid responses were obtained, resulting in an effective response rate of 75.05%. The demographic information of the sample is presented in Table 1: the sample included 170 male and 179 female participants. Among them, 150 were freshmen and sophomores undergraduates, 154 were juniors and seniors undergraduates, 35 were master candidates, and 10 were doctoral candidates. All participants reviewed an informed consent form and completed the survey anonymously. They were provided with a neutral academic study description that emphasized the absence of predetermined answers and encouraged responses based on their genuine experiences and feelings. Participants were free to withdraw from the study at any time. No compensation was provided for participation in this study.

**Table 1:** Basic information of the sample (*N* = 349).

		*N*	%
Gender	Male	170	48.7
	Female	179	51.3
Age	Under 18 years old	13	3.7
	18–21 years old	118	33.8
	21–24 years old	79	22.6
	24–27 years old	85	24.4
	Over 27 years old	54	15.5
	Freshmen and sophomores	150	43
Grade	Juniors and seniors	154	44.1
	Master candidate	35	10
	Doctoral candidate	10	2.9
Daily social media usage	<1 h	42	12
	1–3 h	61	17.5
	3–5 h	128	36.7
	5–7 h	61	17.5
	>7 h	57	16.3

### 3.2 Measure

#### 3.2.1 Personal background information

This section includes basic background information about the participants, such as gender, age, grade, and daily social media usage. Given that these variables may influence the process of online trolling behavior, this study will consider controlling for them.

#### 3.2.2 SNS fatigue questionnaire

We utilized the Chinese version of the SNS Fatigue (SFG) Questionnaire, adapted by Lee et al. (2016) and revised by Li et al. (2018), to measure the subjective and self-assessed level of fatigue experienced by users while using social networking services (SNS) such as WeChat and QQ. The questionnaire consists of six items (e.g., “I find it difficult to relax after continually using SNSs.”) and uses a 5-point Likert scale, where 1 represents “strongly disagree” and 5 represents “strongly agree.” Higher scores indicate a greater degree of social media fatigue. In this study, the scale demonstrated good reliability, with a Cronbach *α* coefficient of 0.933. The confirmatory factor analysis (CFA) of the questionnaire also showed good fit indices: *x^2^*/*df* = 0.494, RMSEA = 0.000, NFI = 0.997, GFI = 0.996, and CFI = 1.000.

#### 3.2.3 Personal relative deprivation scale

We employed the Chinese version of the Personal Relative Deprivation Scale (PRDS), originally developed by Callan et al. (2011) and translated and revised by Peng et al. (2021), to measure the feelings of resentment and dissatisfaction that users experience due to perceived disadvantage when comparing themselves to reference groups while using SNS. The original scale consists of five items (e.g., “I feel deprived when I think about what I have compared to what other people like me have.”) rated on a 6-point Likert scale, where 1 represents “strongly disagree” and 6 represents “strongly agree.” Higher scores indicate a greater level of relative deprivation. Items 2 and 4 are reverse-scored. In the context of Chinese culture, the two reverse-scored items exhibited low primary factor loadings in the original scale; thus, they were removed to create a three-item version, PRDS-3, which demonstrated good item discrimination. In this study, PRDS-3 showed good reliability, with a Cronbach *α* coefficient of 0.835. The confirmatory factor analysis also indicated a good model fit: *x^2^*/*df* = 0.113, RMSEA = 0.000, NFI = 1.000, GFI = 1.000, and CFI = 1.000.

#### 3.2.4 Word sentence association paradigm for hostility

We used the Word Sentence Association Paradigm for Hostility (WSAP-Hostility) developed by Dillon et al. (2016) to assess the tendency of participants to interpret others’ intentions as hostile in ambiguous situations. The scale has demonstrated adequate validity and internal consistency in the Chinese context (Quan and Xia, 2019). It consists of 16 contextually ambiguous sentences (e.g., “Someone is in your way.”), each followed by an adjective related to hostility (e.g., “inconsiderate”). Participants are asked to rate the similarity between the given sentence and the hostility-related adjective. The scale uses a 6-point Likert scale, where 1 represents “not at all similar” and 6 represents “extremely similar.” Higher scores indicate a stronger attributional bias towards hostility. In this study, the scale demonstrated good reliability, with a Cronbach *α* coefficient of 0.974. The confirmatory factor analysis also showed good fit indices: *x^2^*/*df* = 1.210, RMSEA = 0.025, NFI = 0.978, GFI = 0.958, and CFI = 0.996.

#### 3.2.5 Revised global assessment of internet trolling

We employed the revised Global Assessment of Internet Trolling (GAIT-R) developed by Sest and March (2017) to assess online malicious commenting behavior. This self-report measure consists of eight items (e.g., “Although some people think my posts/comments are offensive, I think they are funny.”) and is adapted from the Global Assessment of Internet Trolling (GAIT) scale originally developed by Buckels et al. (2014). The scale uses a 5-point Likert scale, where 1 represents “strongly disagree” and 5 represents “strongly agree.” Higher scores indicate a greater degree of internet trolling behavior, with item 8 being reverse-scored. In this study, the scale demonstrated good reliability, with a Cronbach *α* coefficient of 0.946. The confirmatory factor analysis also indicated good fit indices: *x^2^*/*df* = 1.463, RMSEA = 0.036, NFI = 0.987, GFI = 0.979, and CFI = 0.996.

### 3.3 Data analysis

In this study, the data from participants were analyzed using SPSS 27.0 and Amos 28.0. The empirical analysis followed these steps: First, frequency analysis was conducted using SPSS 27.0 to understand the demographic characteristics of the participants. To meet the research requirements, we re-evaluated the reliability and construct validity of the scales to ensure acceptable reliability and validity within the context of the study sample. For this purpose, confirmatory factor analysis (CFA) and structural equation modeling (SEM) were performed in Amos 28.0 to test the construct validity of the survey, verifying the reliability and validity of the measurement tools within the study’s measurement model. Second, we applied the method of controlling for the effects of an unmeasured latent methods factor (ULMC) to examine the error due to common method bias (CMB) (Williams and McGonagle, 2016). Third, a bivariate correlation analysis was conducted on the variables used in this study to examine the relationships among social media fatigue, relative deprivation, hostile attribution bias, and online trolling behavior. Finally, based on the results of the measurement model, we used Amos 28.0 to construct and analyze the mediation model to test the research hypotheses and examine the mediation effects. The 95% confidence intervals (CIs) for the mediation effects were calculated using the bias-corrected percentile bootstrap method (*N* = 5,000). These effects were considered statistically significant if the confidence intervals did not include zero.

## 4 Results

### 4.1 Common method bias test

During the survey process, we employed anonymous measurements and partial reverse scoring of items to control for common method bias (CMB) (Zhou and Long, 2004). To ensure the validity of the measurement results, a two-factor model was used to test for CMB (Richardson et al., 2009). The results showed that the fit indices for the model without the method factor were as follows: CFI = 1.000, TLI = 1.000, RMSEA = 0.000, SRMR = 0.0163; for the model with the added method factor, the fit indices were: CFI = 1.000, TLI = 1.004, RMSEA = 0.000, SRMR = 0.0137. All changes were below the critical values (ΔCFI <0.10, ΔTLI <0.10, ΔRMSEA <0.05, ΔSRMR <0.05), indicating that the common method bias present in the study is not severe.

### 4.2 Describe statistics and related analysis

Table 2 presents the mean, standard deviation, and Pearson correlation matrix for each variable. The results showed that social media fatigue was significantly positively correlated with online trolling behavior (*r* = 0.575, *p* < 0.01). Both relative deprivation and hostile attribution bias were positively correlated with social media fatigue and online trolling behavior (*r* = 0.270–0.457, *p* < 0.01). Additionally, gender was correlated with relative deprivation and hostile attribution bias (*r* = −0.112, *r* = −0.131, *p* < 0.05), academic year was correlated with hostile attribution bias (*r* = −0.229, *p* < 0.01), and daily social media usage was correlated with relative deprivation, hostile attribution bias, and online trolling behavior (*r* = 0.137, 0.197, 0.178, *p* < 0.05).

**Table 2:** Descriptive statistics and correlations among study variables.

Variables	M	SD	1	2	3	4	5	6	7	8
1. Gender	–	–	1							
2. Age	–	–	0.024	1.000						
3. Grade	–	–	0.021	0.761**	1					
4. DSMU	–	–	−0.016	−0.021	−0.099	1				
5. SMF	3.342	1.158	−0.088	−0.006	−0.029	−0.010	1			
6. RD	4.505	1.387	−0.112*	−0.008	−0.061	0.137*	0.313**	1		
7. HAB	4.049	1.369	−0.131*	0.003	−0.229**	0.197**	0.294**	0.457**	1	
8. OT	3.332	1.126	0.014	−0.050	−0.042	0.178**	0.270**	0.283**	0.270**	1

*N* = 349. *p < 0.05, **p < 0.01. M, mean; SD, standard deviation; DSMU, daily social media usage; RD, relative deprivation; SMF, social media fatigue; 6. HAB, hostile attribution bias; OT, online trolling. Gender was dummy coded with 0 for male participants and 1 for female participants.

### 4.3 Testing for the mediation effect

To control for potential measurement errors arising from multiple items within the scales, we used item parceling. Following the recommendations of Wu and Wen (2011), the single-dimension scales, SNS Fatigue and WSAP-Hostility, were parceled using the factor balance method. The observed variables were replaced by the average scores of each parcel, resulting in three and four latent variable indicators, respectively. For the GAIT-R, a three-dimensional scale, the dimensions were combined into three latent variable indicators using the internal consistency method. Specifically, the factors of Trolling Frequency, Trolling Enjoyment, and Trolling Identity constituted new indicators of the latent variable for online trolling behavior. Due to the PRDS being a single-dimensional scale with a small number of items, the original items were used directly.

Following the mediation effect testing procedure proposed by Wen and Ye (2014), we first built a model in Amos 28.0 to test the association between social media fatigue and online trolling behavior. The results showed a good model fit to the data: *x^2^*/*df* = 0.439, RMSEA = 0.000, NFI = 0.998, GFI = 0.997, and CFI = 1.000. Social media fatigue showed a significant positive association with online trolling behavior (*β* = 0.289, *p* < 0.001), confirming *Hypothesis 1*. The standardized loadings of all observed variables on their corresponding latent variables ranged from 0.896 to 0.933, indicating that the measurement model met the desired standards and that the observed variables adequately reflected their corresponding latent variables, allowing for further testing of the structural model. A structural equation model was constructed with social media fatigue as the independent variable, online trolling behavior as the dependent variable, and relative deprivation and hostile attribution bias as mediating variables. Based on the correlation analysis results, gender, academic year, and daily social media usage were included as control variables in the model. The results indicated that the chain mediation model showed a good fit: *x^2^*/*df* = 1.215, RMSEA = 0.025, NFI = 0.975, GFI = 0.961, and CFI = 0.995.

**Figure 1:**
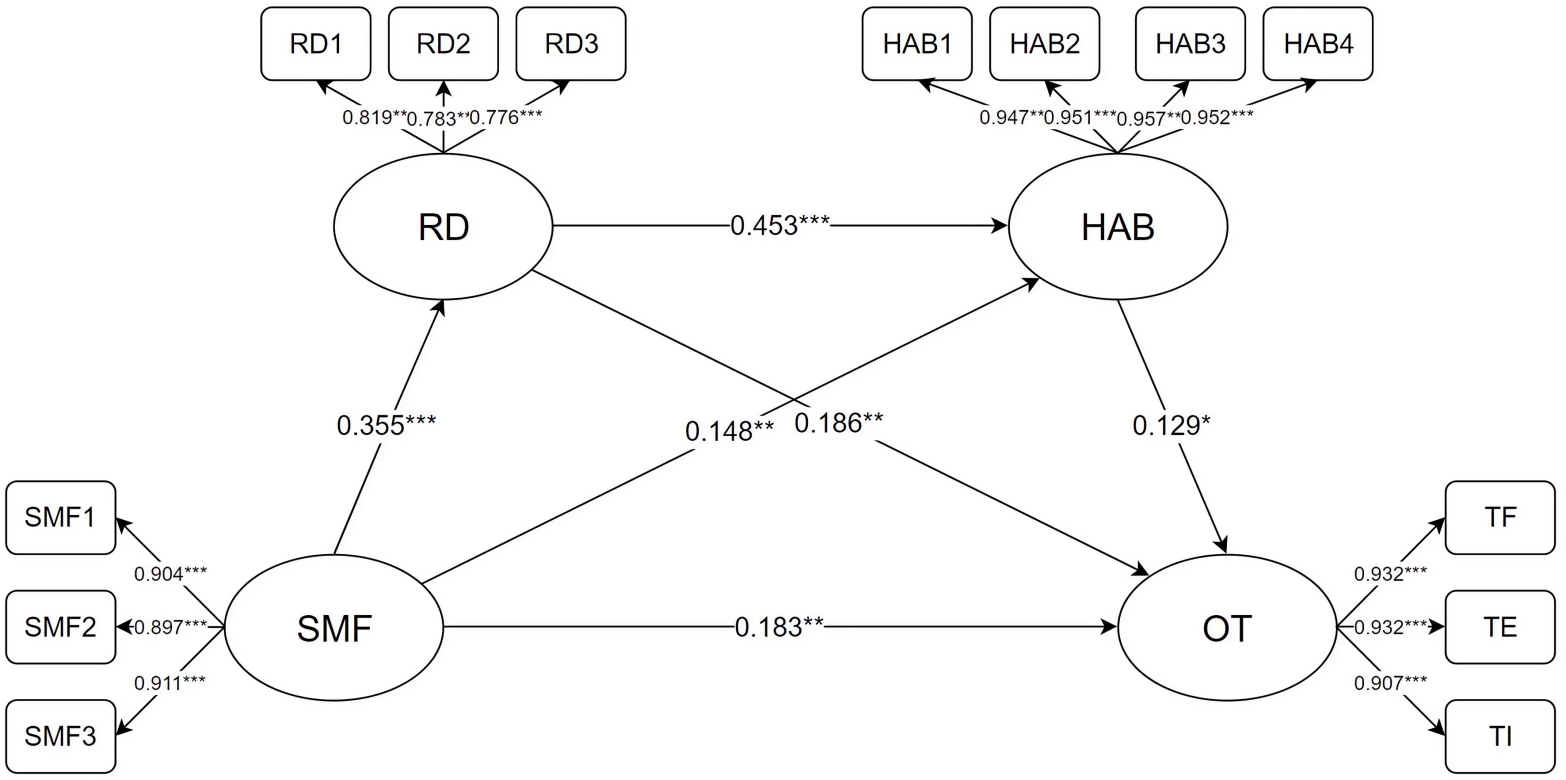
Path analysis diagram and model fit test of social media fatigue, relative deprivation, hostile attribution bias, and online trolling. SMF, social media fatigue; RD, relative deprivation; HAB, hostile attribution bias; OT, online trolling; TF, trolling frequency; TE, trolling enjoyment; TI, trolling identity. Each variable in the model was standardized. *p < 0.05, **p < 0.01, ***p < 0.001.

Given the satisfactory model fit, we used the bias-corrected non-parametric percentile bootstrap method to test the significance of the mediation effects. This procedure involved 5,000 resampling iterations to calculate 95% confidence intervals (CIs). The mediation effect was considered significant if the CIs did not include zero. As shown in Table 3 and Figure 1, the mediating effect of relative deprivation between social media fatigue and online trolling behavior was significant, with a 95% CI of [0.020, 0.132] and an indirect effect of 0.066, confirming *Hypothesis 2*. The mediating effect of hostile attribution bias between social media fatigue and online trolling behavior was also significant, with an indirect effect of 0.019 and a 95% CI of [0.001, 0.054], confirming *Hypothesis 3*. Additionally, the chain mediating effect of relative deprivation and hostile attribution bias between social media fatigue and online trolling behavior was significant, with an indirect effect of 0.021 and a 95% CI of [0.002, 0.046], confirming *Hypothesis 4*. Overall, the total indirect effect of relative deprivation and hostile attribution bias between social media fatigue and online trolling behavior was 0.106, accounting for 36.70% of the total effect (0.289).

**Table 3:** Path and effect decomposition table of social media fatigue on online trolling.

Effect	Path relationship	Effect size	Boostrap SE	Boostrap 95% CI		Relative intermediary effect
				Lower	Upper	
Direct effect	Social media fatigue → online trolling	0.183	0.062	0.062	0.306	63.30%
	Social media fatigue → relative deprivation → online trolling	0.066	0.028	0.020	0.132	22.90%
Indirect effect	Social media fatigue → hostile attribution bias → online trolling	0.019	0.013	0.001	0.054	6.60%
	Social media fatigue → relative deprivation → Hostile attribution bias → online trolling	0.021	0.011	0.002	0.046	7.20%
Total intermediary effect		0.106	0.027	0.057	0.167	36.70%
Total effect		0.289	0.054	0.179	0.390	

SE, standard error; CI, confidence interval. Each variable in the model was standardized.

## 5 Discussion

This study examined the impact of social media fatigue on online trolling behavior, as well as the mediating roles of relative deprivation and hostile attribution bias. The results support our hypotheses, providing new insights into the mechanisms underlying the formation of online trolling behavior.

### 5.1 The impact of social media fatigue on online trolling behavior

Our findings provide empirical support for the application of the General Aggression Model (GAM) in understanding online aggressive behavior. The significant association between social media fatigue and online trolling behavior aligns with GAM’s proposition about how situational factors relate to aggressive outcomes. This result is consistent with previous research, such as Wu et al., who found a positive correlation between social media fatigue and online aggressive behavior (Wu et al., 2022). Through the lens of GAM, social media fatigue can be understood as a situational factor that relates to internal states in several ways. Prolonged immersion in social media is associated with information overload and social pressure, which show relationships with increased fatigue (Lee et al., 2016). This negative state correlates with the adoption of maladaptive coping strategies, such as posting aggressive comments (Zhang et al., 2012). Moreover, social media fatigue may diminish users’ self-control capacity, making them more susceptible to impulsive behavior. This is consistent with the ego depletion theory proposed by Baumeister et al., which posits that an individual’s self-control resources are finite, and prolonged social media use may deplete these resources, resulting in subsequent self-control failures (Baumeister et al., 2007).

The causal direction between social media fatigue and online trolling behavior remains a topic worthy of further exploration. Although current research generally treats social media fatigue as a precursor to trolling behavior, the possibility of reverse causality—where users engaging in trolling behavior exacerbate their level of social media fatigue—cannot be completely ruled out. However, considering that social media fatigue is characterized as a progressively accumulated psychological state, it is more likely to serve as a cause of trolling behavior rather than its result. This judgment is based on the following reasons: First, in online interaction environments, there is an essential distinction between the individuals who engage in trolling behavior and those who bear its impact (including direct victims and bystanders). According to the research by Craker and March, trolling behavior often arises from momentary emotional impulses, such as anger, a desire for entertainment, or the need to satisfy social cravings, rather than from long-term accumulated exhaustion (Craker and March, 2016). Silver further points out that fatigue primarily affects observers and victims of such behavior, rather than the initiators (Silver, 2006). Second, social media fatigue is a complex state of psychological exhaustion. As described by Ravindran et al., it is the result of prolonged exposure to factors such as information overload, social pressure, and the challenges of managing virtual identities (Ravindran et al., 2014). In contrast, trolling behavior is typically characterized by its sudden onset and situational dependence. From this perspective, viewing fatigue as a trigger for trolling behavior aligns better with theoretical logic.

This inference is further supported by the stressor-strain-outcome (SSO) theoretical framework. According to Malik et al., social media fatigue depletes an individual’s psychological resources, increases hostility, and thereby raises the likelihood of engaging in aggressive behavior (Malik et al., 2021). More compellingly, Aalbers et al., through time-series analysis, found that fatigue states effectively predict subsequent social media behavior patterns, whereas evidence for reverse causality is relatively weak (Aalbers et al., 2019).

### 5.2 The mediating role of relative deprivation

The mediating role of relative deprivation aligns with GAM’s affective pathway. Our findings suggest that the relationship between social media fatigue and online trolling behavior operates in part through emotional states, specifically feelings of relative deprivation. This is consistent with GAM’s proposition that aggressive behavior is associated with negative emotional states that arise from situational factors. In the context of social media, our findings reveal how platform-induced fatigue correlates with heightened feelings of relative deprivation, which in turn shows associations with aggressive online behavior.

The study found that relative deprivation partially mediates the relationship between social media fatigue and online trolling behavior. This finding aligns with the research of Greitemeyer and Sagioglou, who discovered a significant positive correlation between relative deprivation and aggressive behavior (Greitemeyer and Sagioglou, 2017). Through the theoretical lens of GAM, social media platforms create environments where emotional responses play a crucial role in behavioral outcomes. When users experience social media fatigue, the platforms’ inherent features for social comparison become particularly salient. This is evidenced by Verduyn et al.’s finding that frequent social media use correlates with increased opportunities for social comparison, potentially exacerbating users’ sense of relative deprivation (Verduyn et al., 2017).

Our results further indicate that the relationship between social media fatigue and relative deprivation may be particularly pronounced in the social media context. Vogel et al. found that the frequency of social media use negatively correlates with social comparison and self-esteem levels (Vogel et al., 2014). When users perceive themselves to be in a disadvantaged position, this correlates with increased likelihood of engaging in aggressive behaviors as a potential means of emotional regulation or self-esteem maintenance (Smith et al., 2012). This pattern is further supported by Appel et al.’s meta-analysis, which revealed significant associations between social media use and feelings of envy (Appel et al., 2016). These emotional experiences may intensify users’ sense of relative deprivation, showing stronger relationships with aggressive behavioral tendencies.

Furthermore, viewing these findings through GAM’s framework helps explain why relative deprivation serves as an effective mediating mechanism. The model suggests that emotional states do not merely co-occur with aggressive behavior but serve as critical internal processes that help shape behavioral responses. In the social media context, this means that fatigue-induced relative deprivation may influence how users process and respond to social information. Yang et al.’s research supports this interpretation, showing that social media-induced envy correlates with increased depressive symptoms (Yang et al., 2018), which in turn could further exacerbate aggressive behavior.

### 5.3 The mediating role of hostile attribution bias

The mediating role of hostile attribution bias provides empirical support for GAM’s cognitive pathway. Our results demonstrate how cognitive processing patterns, particularly the tendency to interpret ambiguous situations as hostile, serve as a mechanism in the relationship between social media fatigue and trolling behavior. This finding extends GAM’s application to the online context, where the absence of face-to-face social cues may amplify the role of cognitive biases in shaping behavioral responses.

Our results indicate that hostile attribution bias partially mediates the relationship between social media fatigue and online trolling behavior. This finding aligns with the longitudinal study by Zhu et al., which discovered that hostile automatic thoughts mediate the relationship between daily exposure to violence and online aggressive behavior (Zhu et al., 2023). Through GAM’s theoretical framework, we can understand how social media fatigue correlates with cognitive biases: when users experience fatigue, they may show increased tendencies to interpret ambiguous online interactions through a hostile lens (Dodge, 2006). This hostile attribution bias may further provoke aggressive responses in users, increasing the likelihood of online trolling behavior (Orobio de Castro et al., 2002).

The significance of hostile attribution bias in the online context can be further understood through GAM’s emphasis on cognitive processing. Chen et al. found a significant positive correlation between hostile attribution bias and cyberbullying behavior among adolescents (Chen et al., 2017). Additionally, research by Kokkinos and Voulgaridou supports this notion, revealing that hostile attribution bias shows significant associations not only with traditional bullying but also with cyberbullying behavior, suggesting the robustness of GAM’s cognitive pathway across different contexts (Kokkinos and Voulgaridou, 2017).

Notably, the unique characteristics of the social media environment may exacerbate the impact of hostile attribution bias. The absence of non-verbal cues in face-to-face communication makes it easier for users to misinterpret others’ intentions (Kiesler et al., 1984). Through GAM’s framework, this increased ambiguity may strengthen the relationship between cognitive biases and aggressive responses. The social information processing-cyber framework proposed by Runions et al. provides additional support for this interpretation, emphasizing how online environments may amplify the impact of hostile attribution bias on behavioral outcomes (Runions et al., 2017).

### 5.4 The chain mediating effect of relative deprivation and hostile attribution bias

The observed sequential mediation effect of relative deprivation and hostile attribution bias provides empirical support for GAM’s integrated process model. Our findings suggest that affective and cognitive pathways do not operate in isolation, but rather show interconnected associations, as proposed by GAM. Specifically, social media fatigue appears to first trigger affective responses (relative deprivation), which then influence cognitive processing patterns (hostile attribution bias), ultimately increasing the likelihood of aggressive behavior (online trolling). This sequential process aligns with GAM’s proposition that internal states interact dynamically rather than functioning as independent channels (Anderson and Bushman, 2002).

This chain mediation pattern is supported by recent research demonstrating the interconnected nature of emotional and cognitive processes in aggressive behavior. Guo et al. found that relative deprivation predicts changes in displaced aggression through the sequential mediation of hostile attribution bias and moral disengagement, highlighting how deprivation-related emotions can shape cognitive interpretations (Guo et al., 2024). The sequential nature of these processes is further evidenced by studies examining cognitive mechanisms in aggression development. Su et al. demonstrated that hostile attribution bias mediates the relationship between interpersonal traits and displaced aggressive behavior (Su et al., 2021), while Quan et al. found that hostile attribution bias and anger rumination serve as sequential mediators between trait anger and reactive aggression (Quan et al., 2021). These findings aligns with Cen et al., who found that hostile rumination and moral disengagement serve as chain mediators between self-control and reactive-proactive aggression (Cen et al., 2022). Otherwise, a longitudinal study by Pabian and Vandebosch revealed that social anxiety indirectly predicts subsequent cyberbullying behavior by increasing hostile attribution bias (Pabian and Vandebosch, 2016), providing additional support for our chain mediation model.

The chain mediation identified in our study particularly highlights how GAM’s affective and cognitive routes may amplify each other in the digital context. When users experience social media fatigue, the resulting sense of relative deprivation appears to create an emotional context that makes hostile interpretations more likely. This emotional-cognitive interaction then heightens the probability of aggressive online behavior, demonstrating the interconnected nature of internal processes proposed by GAM.

From an existential perspective, social media fatigue and online trolling behavior reflect the profound existential dilemmas faced by modern individuals in the digital age. These phenomena reveal the contradictions and struggles between the virtual and the real, freedom and responsibility, meaning and nihilism. Social media platforms provide users with opportunities to construct virtual identities, but the discrepancy between these virtual identities and authentic selves may lead to a sense of internal fragmentation. Social media fatigue can be understood as a response to this fragmentation, a weariness resulting from the constant oscillation between the virtual and the real. Simultaneously, online trolling behavior may represent an extreme rebellion against virtual identities, a distorted attempt to seek a sense of “authentic” existence (Sartre, 1956; Dreyfus, 2008).

### 5.5 Limitations and directions for future research

The present investigation, while illuminating the intricate relationships between social media fatigue, relative deprivation, and hostile attribution bias in online trolling behavior through GAM’s theoretical framework, encompasses several methodological and conceptual constraints that warrant consideration. The primary limitation stems from our cross-sectional research design, which, despite yielding valuable insights through GAM’s application, precludes definitive causal inferences and comprehensive capture of the model’s proposed dynamic processes. To address this constraint, subsequent research endeavors might benefit from employing longitudinal methodologies, particularly the Experience Sampling Method, to elucidate the temporal dynamics between variables and examine how situational factors like social media fatigue shape users’ internal states and behavioral manifestations in their daily experiences (Csikszentmihalyi and Larson, 1987).

Our investigation, while successfully examining cognitive mechanisms through hostile attribution bias and affective pathways via relative deprivation within GAM’s framework, did not encompass certain crucial mediating variables, notably physiological arousal. Moreover, though demographic factors were controlled, the study did not extensively investigate stable personality characteristics that GAM suggests might moderate the relationship between situational inputs and aggressive behavioral outcomes. The study’s generalizability faces potential constraints due to its sample composition, predominantly comprising Chinese university students. Cultural variations and age-related differences in social media utilization and psychological responses necessitate validation across more diverse populations. Additionally, the reliance on self-reported data introduces potential methodological concerns regarding social desirability bias and recall accuracy. Future investigations could strengthen methodological rigor by incorporating experimental designs and diary studies to obtain more objective measurements.

While our research highlighted the mediating roles of relative deprivation and hostile attribution bias within GAM’s framework, future investigations could expand the theoretical scope by incorporating additional mediating variables, such as emotion regulation capabilities and self-control mechanisms. This expansion would foster a more comprehensive understanding of the relationship between social media fatigue and trolling behavior. The findings of Erreygers et al., demonstrating emotion regulation’s moderating effect on the relationship between social media use and cyberbullying, suggest promising directions for future GAM applications in online contexts (Erreygers et al., 2018).

The application of GAM to digital environments raises intriguing theoretical questions about the model’s adaptability to cyber-aggression’s unique characteristics. Future research should investigate whether GAM’s proposed cyclical processes—particularly the feedback loop between aggressive outcomes and subsequent situational and personal factors—manifest differently in digital versus face-to-face interactions. While GAM posits that aggressive behavior emerges from both immediate situational triggers and the development of aggressive knowledge structures over time, our cross-sectional methodology could not capture how sustained social media fatigue might contribute to the formation of hostile attribution patterns and subsequent stable trolling behaviors. Longitudinal investigations would be particularly valuable in examining these developmental trajectories.

## 6 Conclusion

This study investigated the complex relationships among social media fatigue, relative deprivation, hostile attribution bias, and online trolling behavior through the theoretical lens of the General Aggression Model (GAM). Through a questionnaire survey of 349 Chinese college students, the research found that social media fatigue significantly positively association with online trolling behavior, with relative deprivation and hostile attribution bias playing crucial mediating roles in this process. Specifically, social media fatigue shows both direct and indirect associations with online trolling behavior, with the indirect relationships operating through relative deprivation and hostile attribution bias. These findings elucidate the psychological mechanisms underlying the formation of online trolling behavior, enriching the theoretical foundation of online behavior research while providing new insights for practical interventions. The results suggest that reducing social media fatigue, lowering users’ sense of relative deprivation, and fostering positive attribution styles may be effective approaches to preventing online trolling behavior.

## Data Availability

The raw data that support the findings of this study are available from the corresponding author upon reasonable request.
